# Nrf2 impacts cellular bioenergetics by controlling substrate availability for mitochondrial respiration

**DOI:** 10.1242/bio.20134853

**Published:** 2013-06-20

**Authors:** Kira M. Holmström, Liam Baird, Ying Zhang, Iain Hargreaves, Annapurna Chalasani, John M. Land, Lee Stanyer, Masayuki Yamamoto, Albena T. Dinkova-Kostova, Andrey Y. Abramov

**Affiliations:** 1 Department of Molecular Neuroscience, UCL Institute of Neurology, London WC1N 3BG, UK; 2 Jacqui Wood Cancer Centre, Division of Cancer Research, Medical Research Institute, University of Dundee, Dundee DD1 9SY, UK; 3 Neurometabolic Unit, National Hospital, Queen Square, London WC1N 3BG, UK; 4 Department of Medical Biochemistry, Tohoku University Graduate School of Medicine, 2-1 Seiryo-cho, Aoba-ku, Sendai 980-8575, Japan; 5 Department of Pharmacology and Molecular Sciences, Johns Hopkins University School of Medicine, Baltimore, MD 21205, USA

**Keywords:** Nrf2, Keap1, Energy metabolism, Oxidative phosphorylation, Mitochondria

## Abstract

Transcription factor Nrf2 and its repressor Keap1 regulate a network of cytoprotective genes involving more than 1% of the genome, their best known targets being drug-metabolizing and antioxidant genes. Here we demonstrate a novel role for this pathway in directly regulating mitochondrial bioenergetics in murine neurons and embryonic fibroblasts. Loss of Nrf2 leads to mitochondrial depolarisation, decreased ATP levels and impaired respiration, whereas genetic activation of Nrf2 increases the mitochondrial membrane potential and ATP levels, the rate of respiration and the efficiency of oxidative phosphorylation. We further show that Nrf2-deficient cells have increased production of ATP in glycolysis, which is then used by the F_1_F_o_-ATPase for maintenance of the mitochondrial membrane potential. While the levels and *in vitro* activities of the respiratory complexes are unaffected by Nrf2 deletion, their activities in isolated mitochondria and intact live cells are substantially impaired. In addition, the rate of regeneration of NADH after inhibition of respiration is much slower in Nrf2-knockout cells than in their wild-type counterparts. Taken together, these results show that Nrf2 directly regulates cellular energy metabolism through modulating the availability of substrates for mitochondrial respiration. Our findings highlight the importance of efficient energy metabolism in Nrf2-mediated cytoprotection.

## Introduction

To adapt and survive, eukaryotic organisms have evolved an elaborate network of cytoprotective proteins (e.g. NAD(P)H:quinone oxidoreductase 1, glutathione transferases, γ-glutamylcysteine ligase, heme oxygenase 1). Functionally, they are extraordinarily versatile and, by mechanisms which include direct antioxidant activity, obligatory 2-electron reduction reactions, conjugation with endogenous ligands, recognition, repair and removal of damaged proteins, efficiently detoxify numerous endogenous and exogenous damaging agents, and facilitate their elimination (Baird and Dinkova-Kostova, 2011; Kensler et al., 2007; Nguyen et al., 2009; Talalay, 2000). These cytoprotective proteins share common transcriptional regulation, through the Keap1-Nrf2 pathway. Under basal conditions Kelch-like ECH-associated protein 1 (Keap1) binds and targets transcription factor Nuclear factor E2-related factor 2 (Nrf2) for ubiquitination and proteasomal degradation via association with Cullin 3-based E3 ubiquitin ligase. Inducers of this pathway react and chemically modify specific cysteine residues of Keap1, which consequently loses its ability to target Nrf2 for degradation. This leads to increased stabilization of Nrf2, its nuclear translocation, and ultimately to coordinate expression of cytoprotective genes. Deletion of Nrf2 renders cells and animals much more sensitive to the damaging effects of electrophiles, oxidants, and inflammatory agents in comparison to their wild-type counterparts; conversely, pharmacological or genetic activation of Nrf2 has protective effects in numerous models of chronic disease (Hayes et al., 2010). Thus, the ability to upregulate Nrf2-dependent genes is crucial for adaptation and survival.

Because of its highly effective and comprehensive cytoprotective role against the consequences of oxidative stress and inflammation, the underlying causes for essentially all chronic degenerative diseases, Nrf2 is now widely recognized as a drug target (Crunkhorn, 2012). Indeed, several activators of Nrf2 have been or currently are in clinical trials. Bardoxolone methyl treatment has been used in advanced chronic kidney disease associated with type 2 diabetes (Pergola et al., 2011a; Pergola et al., 2011b), while dimethyl fumarate has been tested in patients with relapsing-remitting multiple sclerosis (Gold et al., 2012). In human subjects exposed to aflatoxins and air-borne toxins, standardized preparations of broccoli sprouts as sources of the Nrf2 activator sulforaphane or its precursor, glucoraphanin, have shown enhanced detoxification of these environmental toxins, which may in turn lower the health risks associated with such exposures (Kensler et al., 2013).

Emerging experimental evidence suggests that Nrf2 not only activates antioxidant, drug metabolizing, and anti-inflammatory genes in response to stress, but also influences primary metabolism and bioenergetics. Thus, the expression of malic enzyme and three of the enzymes of the pentose phosphate pathway (namely, glucose-6-phosphate dehydrogenase, transaldolase, and transketolase), is severely decreased in the absence of Nrf2 (Lee et al., 2003a; Lee et al., 2003b; Mitsuishi et al., 2012; Thimmulappa et al., 2002; Wu et al., 2011). Together, the catalytic power of these enzymes provides precursors for the biosynthesis of basic metabolites such as reducing equivalents (e.g. NADPH) for the maintenance of glutathione in its reduced state and for the fatty acid biosynthesis, ribose-5-phosphate for the biosynthesis of nucleotides, and erythrose-4-phosphate for the biosynthesis of aromatic amino acids. Most recently, it was shown that Nrf2 redirects glucose and glutamine into anabolic pathways, especially under conditions of sustained activation of the PI3K-AKT signaling pathway (Mitsuishi et al., 2012). This function of Nrf2, together with the fact that upregulation of its target genes lowers oxidative stress and inflammation, can not only protect against the onset of disease, including cancer, but under certain conditions, such as those occurring during constitutive K-Ras and B-Raf signaling, may also favor the growth of established tumors (DeNicola et al., 2011).

Curiously, oxygen consumption and ATP production are decreased following siRNA-mediated Nrf2 knockdown in colon cancer cells (Kim et al., 2011), but the underlying reasons for this observation are not known. Because Nrf2 is a transcription factor implicated in the expression of more than 500 genes (Malhotra et al., 2010), one possibility is that it directly affects the expression of genes coding for protein subunits that comprise the mitochondrial respiratory chain. Alternatively, Nrf2 may control indirectly the function of these proteins by regulating the availability of their substrates. To test these hypotheses, we used a panel of live cell imaging techniques and biochemical enzymatic assays to assess mitochondrial function in primary neuronal cultures, mouse embryonic fibroblasts (MEFs) as well as isolated mitochondria from WT, Nrf2 knockout (KO) and Keap1 KO or knockdown (KD) mice. Strikingly, we found that, in the absence of Nrf2, the mitochondrial function is impaired, whereas under condition of constitutive activation of Nrf2, the mitochondrial function is enhanced. Importantly, some of the phenotypic changes associated with Nrf2 deficiency can be rescued by the addition of substrates for the mitochondrial respiration, suggesting a previously unrecognized role for the Keap1-Nrf2 pathway in the control of substrate availability. These findings highlight both the novel role for the pathway in directly modulating mitochondrial metabolism as well as the importance of mitochondrial metabolism in Nrf2-mediated cytoprotection.

## Results

### Nrf2 affects the mitochondrial membrane potential

To establish the role of Nrf2 in cellular energy metabolism, we characterized the effects of this transcription factor on the mitochondrial membrane potential, respiration, ATP production, and redox homeostasis, as well as on the activity of the mitochondrial complexes using mitochondria, cells, and tissues isolated from wild-type (WT), Nrf2-knockout (Nrf2-KO), Keap1-knockout (Keap1-KO), or Keap1-knockdown (Keap1-KD) mice. Nrf2-KO mice have low and uninducible levels of Nrf2-target proteins (Itoh et al., 1997). Conversely, Keap1-KO mice have constitutively high levels of Nrf2 and its downstream target proteins; however, they die postnatally due to esophageal hyperkeratosis (Wakabayashi et al., 2003). In contrast, Keap1-KD mice are viable: they carry a floxed allele of the *keap1* gene, which reduces its expression and consequently increases the levels of Nrf2, and thus represent a genetic model for constitutive Nrf2 activation (Taguchi et al., 2010).

**Fig. 1.**
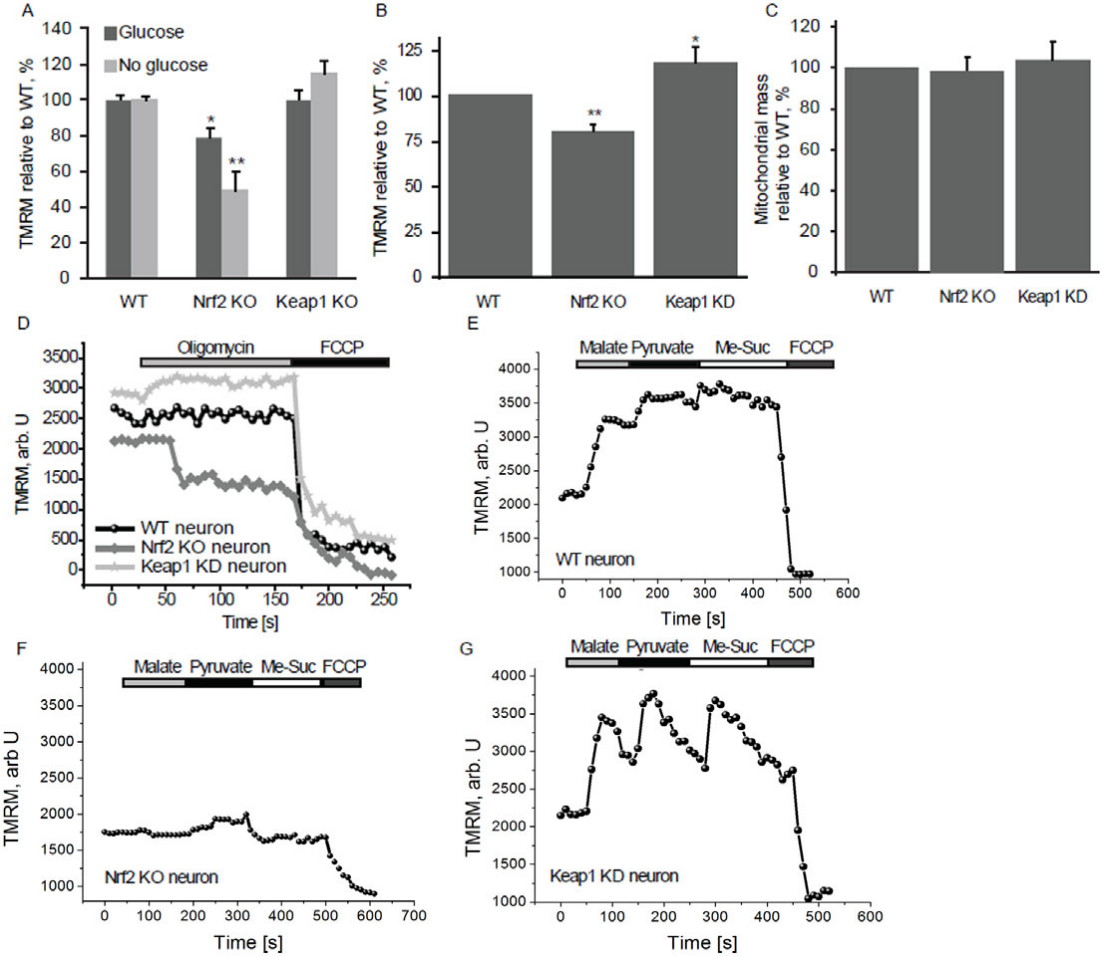
Nrf2 affects the mitochondrial membrane potential. (**A**) Mitochondrial membrane potential in WT, Nrf2-KO and Keap1-KO MEFs in the presence or absence of glucose determined by TMRM fluorescence. (**B**) Mitochondrial membrane potential in primary cortical neurons from WT, Nrf2-KO and Keap1-KD mice. (**C**) Relative mitochondrial mass in Nrf2-KO and Keap1-KD cortical neurons compared to WT cells, measured as the co-localisation of TMRM with the cytosolic calcium dye fluo-4. (**D**) TMRM fluorescence in WT, Nrf2-KO and Keap1-KD cortical neurons after addition of oligomycin (2 µg/ml) and FCCP (0.5 µM). (**E–G**) TMRM fluorescence after sequential addition of malate, pyruvate and methyl succinate (5 mM each) in WT (E), Nrf2-KO (F) and Keap1-KD (G) cortical neurons. FCCP was added in the end to fully depolarise the mitochondria. Data presented as mean percentage compared to WT ± SEM *P<0.05 **P<0.001.

Using live cell imaging, we began by measuring the mitochondrial membrane potential (Δψ_m_), a universal indicator of mitochondrial health and the metabolic state of the cell. Compared to WT, Nrf2-KO mouse embryonic fibroblasts (MEFs) have significantly reduced basal Δψ_m_ (79.2±5.1% of control, *n*>400; *P*<0.05) (assessed by the fluorescent probe tetramethylrhodamine methylester, TMRM); this difference in Δψ_m_ was further accentuated (to 49.7±10.5% of control, *n*>400; *P*<0.001) in the absence of glucose (Fig. 1A). Although not statistically significant, Δψ_m_ was slightly higher in Keap1-KO compared to WT cells (Fig. 1A, *n*>400). Further investigations using primary cortical neurons confirmed the findings in MEFs: Δψ_m_ in Nrf2-KO was 79.8±4.1% of control cells (*n* = 58; *P*<0.001) and 117.9±7.3% in Keap1-KD cells (*n* = 47; *P*<0.05; Fig. 1B). Importantly, there was no difference in the relative mitochondrial mass in neurons from the different genotypes (*n* = 53; Fig. 1C).

In healthy cells the mitochondrial respiratory chain maintains the Δψ_m_. In the event of damage or inhibition of respiration, cells may maintain Δψ_m_ by ATP hydrolysis through the F_1_F_o_-ATPase, thus restoring the proton gradient across the membrane. Application of oligomycin, an inhibitor of the F_1_F_o_-ATPase, to WT midbrain neurons (*n* = 44) induced no response or a slight hyperpolarisation as proton entry through the F_1_F_o_-ATPase was inhibited, as was the case in Keap1-deficient cells (*n* = 53) (Fig. 1D). In contrast, oligomycin (2 µg/ml) caused marked mitochondrial depolarisation in Nrf2-KO neurons (41.6±2.7% decrease in TMRM signal, *n* = 62; Fig. 1D), suggesting that the Δψ_m_ is largely maintained by the hydrolysis of ATP by the F_1_F_o_-ATPase, rather than by respiration.

The differences in basal Δψ_m_ among the genotypes suggest that Nrf2 deficiency could change the activity of the mitochondrial electron transport chain. To test this possibility, we applied to primary cortical neurons substrates for the TCA cycle (malate/pyruvate) which in turn increase the production of the complex I substrate, NADH. We then also added methyl succinate, a substrate for complex II. As expected, in WT cells the sequential addition of malate, pyruvate, and methyl succinate caused a stepwise increase in TMRM fluorescence (Fig. 1E, *n* = 58). Interestingly, the magnitude of the effect of pyruvate and methyl succinate was much greater in Keap1-KD neurons (by 21±0.2% for malate, by 34.5±2.3% for pyruvate, and by 68.4±5.8% for methyl succinate) (Fig. 1G, *n* = 44). Most strikingly, the shape of the response was different. The TMRM fluorescence rapidly increased in response to each substrate; however, it also quickly decreased, suggesting an unusually fast substrate consumption. Notably, these results are consistent with the much lower (by 50–70%) levels of malate, pyruvate and succinate that were observed after a 1-h pulse of [U-^13^C_6_] glucose in Keap1-KO compared to WT MEFs (Mitsuishi et al., 2012). In sharp contrast to WT and Keap1-KD cells, application of both malate and methyl succinate induced a slight depolarisation in Nrf2-KO neurons, and only pyruvate was able to hyperpolarise the mitochondria in these cells (Fig. 1F, *n* = 61).

### Nrf2 regulates respiration

**Fig. 2.**
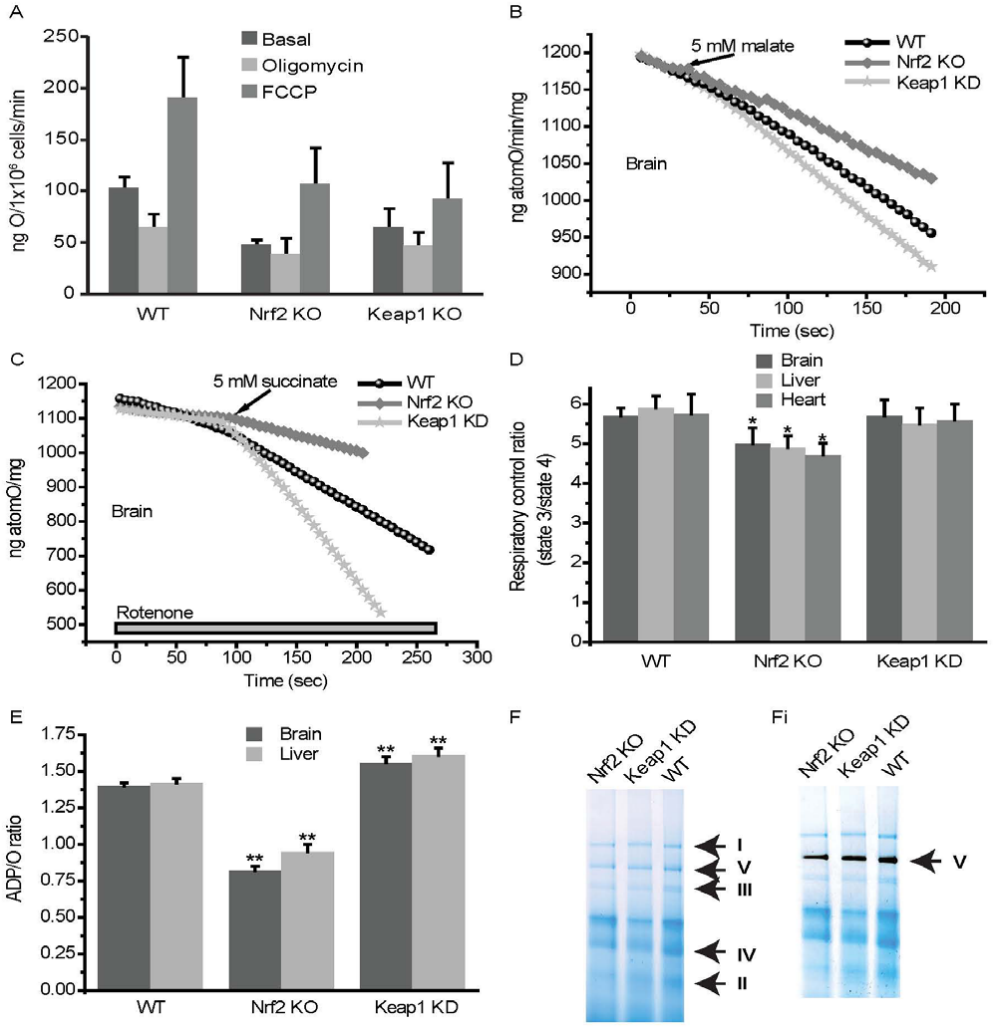
Nrf2 affects mitochondrial respiration. (**A**) Oxygen consumption in WT, Nrf2-KO and Keap1-KO MEF cells under basal conditions or in the presence of oligomycin (2 µg/ml) or FCCP (1 µM). State 4 respiration in isolated brain mitochondria from WT, Nrf2-KO and Keap1-KD mice in the presence of 5 mM malate (**B**) or 5 mM succinate and rotenone (5 µM) (**C**). (**D**) Respiratory control ratio, state 3 respiration (ADP stimulated) to state 4 respiration (no ADP present), in isolated mitochondria from brain, heart and liver of WT, Nrf2-KO and Keap1-KD mice. (**E**) Ratio of ADP to oxygen in isolated mitochondria from brain and liver of WT, Nrf2-KO and Keap1-KD mice. Data presented as mean ± SEM *P<0.05 **P<0.001. (**F**) Coommassie stain of isolated mitochondria, identifying mitochondrial respiratory chain complexes I, II, III, IV and V (F_1_F_o_-ATPase). (Fi) F_1_F_o_-ATPase activity, as seen by a grey precipitate at the level of Complex V.

Mitochondrial respiration is dependent on the Δψ_m_. We therefore evaluated the effect of Nrf2 on oxygen consumption in intact cells and isolated mitochondria. Compared to WT, oxygen consumption in Nrf2-KO and Keap1-KO MEFs was reduced by ∼50% and ∼35%, respectively at the basal state (Fig. 2A, basal). Inhibition of the F_1_F_o_-ATPase by oligomycin decreased respiration by 40% in WT cells, but this effect was attenuated in both Nrf2-KO and Keap1-KO cells, with Nrf2-KO cells showing almost complete insensitivity to oligomycin (Fig. 2A, oligomycin). Upon addition of the uncoupler FCCP, the oxygen consumption of WT cells nearly doubled, (Fig. 2A, FCCP). The effect of FCCP was similar in Nrf2-KO, but was diminished in Keap1-KO cells (Fig. 2A).

To investigate the differences in respiration in detail, we isolated mitochondria from brain and liver of WT, Nrf2-KO and Keap1-KD animals. The rate of oxygen consumption in state 4 respiration was dependent on substrates and it was observed in mitochondria of different tissue origin. Thus, malate induced a higher rate of oxygen consumption in Keap1-KD mitochondria compared to WT (2.3-fold increase in rate compared to WT; *n* = 3, *P*<0.0001) and had a significantly smaller effect on Nrf2-KO mitochondria (2.1-fold decrease in rate compared to WT; *n* = 3, *P*<0.0001; Fig. 2B). Application of Complex II substrate (succinate in the presence of rotenone; Fig. 2C) also activated oxygen consumption more strongly in Keap1-KD (2.7-fold increase in rate compared to WT; *n* = 3, *P*<0.0001) compared to WT mitochondria; its effect was opposite in Nrf2-KO (2.9-fold decrease in rate compared to WT; *n* = 3, *P*<0.0001). Stimulation of mitochondria with glutamate or pyruvate had similar effects on WT, Keap1-KD and Nrf2-KO mitochondria (not shown). The respiratory control ratio (RCR), the ratio of state 3 (ADP-stimulated) to state 4 respiration (no ADP present), is an indication of the degree of coupling of the mitochondrial respiratory chain activity to oxidative phosphorylation (Chance and Williams, 1955). The RCR was decreased in Nrf2-KO mitochondria, while Keap1-KD showed almost no difference compared to WT, indicating that the higher rate of respiration in Keap1-KD mitochondria is not due to uncoupling of oxidative phosphorylation (Fig. 2D). The efficiency of oxidative phosphorylation in mitochondria can be estimated as a ratio of ADP to oxygen consumed for ATP synthesis. Nrf2 deficiency significantly decreased oxidative phosphorylation efficiency in brain and liver mitochondria, which could be due to inhibited respiration and mild uncoupling. Conversely, Keap1-KD mitochondria had a significantly higher ADP/O ratio suggesting that oxidative phosphorylation in these mitochondria is more efficient than in WT (Fig. 2E). Importantly, the differences in respiration among the genotypes are not due to alterations in the levels of the enzymes in the respiratory chain as the activities of the mitochondrial Complex I, II and IV as well as the F_1_F_o_-ATPase in brain tissue isolated from animals of the three genotypes, are very similar (Table 1; Fig. 2F).

**Table 1.**
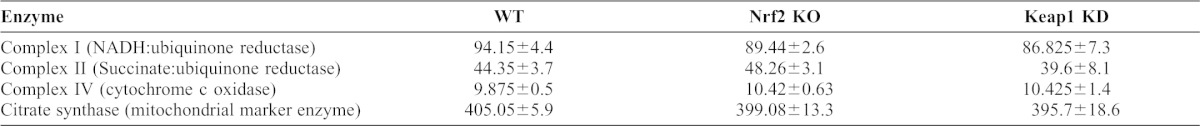
Activities of respiratory chain enzymes in brain tissue isolated from WT, Nrf2 KO, and Keap1 KD 8-week-old male mice (*n* = 4). All activities except complex IV (k/min/mg) are expressed as nmol/min/mg. Values are means ± SEM.

### Nrf2 affects the production of ATP

**Fig. 3.**
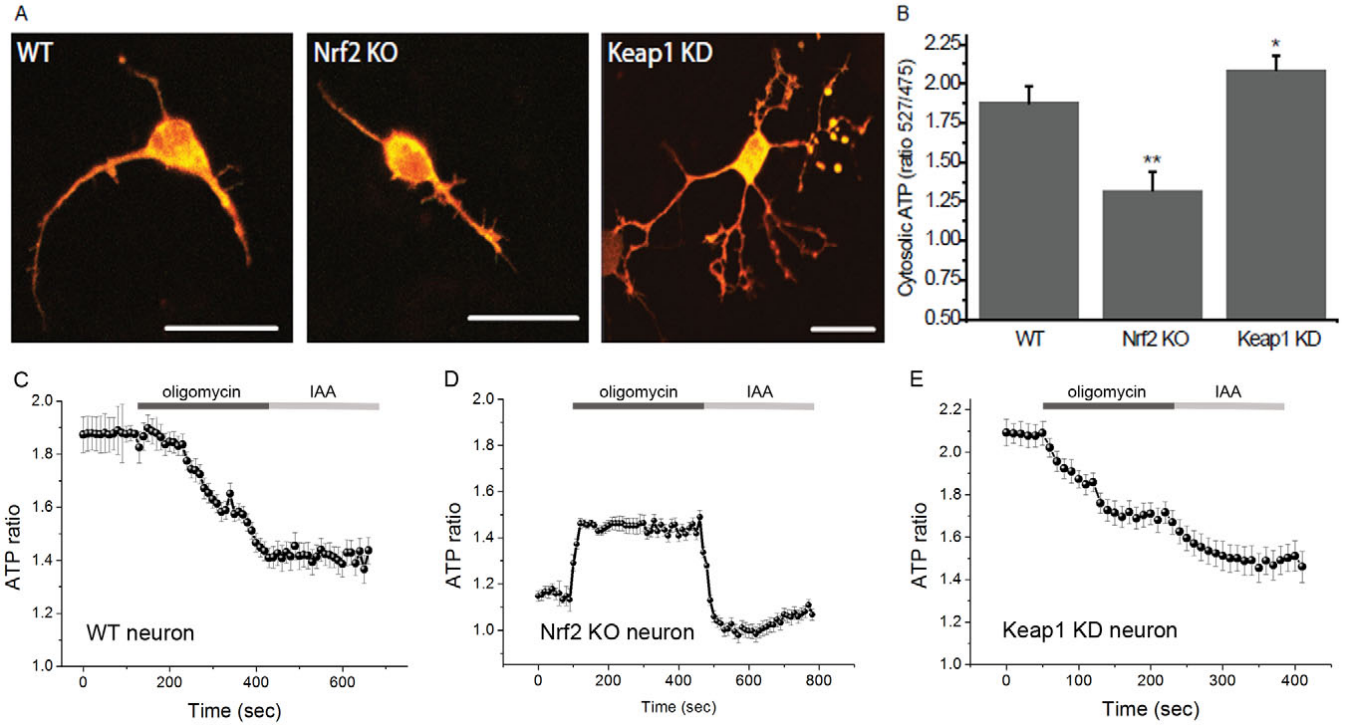
Nrf2 modulates ATP production. (**A**) Cytosolic ATP in primary cortical WT, Nrf2-KO and Keap1-KD neurons transfected with AT 1.03, visualised as a merge of fluorescence images at 527 nm (red) and 475 nm (green). Scale bars: 20 µm. (**B**) Quantification of basal levels of cytosolic ATP in primary cortical WT, Nrf2-KO and Keap1-KD neurons. Data presented as mean ± SEM *P<0.05 **P<0.001. (**C–E**) ATP levels as fluorescence as the ratio 527/475 nm in WT (C), Nrf2-KO (D) and Keap1-KD (E) neurons after inhibition of oxidative phosphorylation by oligomycin (2 µg/ml) and glycolysis by iodoacetic acid (IAA – 20 µM).

The reduced respiration and its insensitivity to oligomycin in Nrf2-KO cells indicate that oxidative phosphorylation could be impaired in the absence of Nrf2. We therefore compared the ATP levels in neurons of the different genotypes (Fig. 3A), and investigated the impact of oxidative phosphorylation (estimated by inhibition with oligomycin) or glycolysis (estimated by inhibition with iodoacetic acid – IAA). Compared to WT, the basal level of ATP in Keap1-KD cells was significantly higher (by 14.6±0.9%, *n* = 36; *P*<0.05), whereas it was lower (by 32.9±2.3%, *n* = 41; *P*<0.001) in Nrf2-KO cells (Fig. 3B). More importantly, the use of inhibitors revealed that the mechanism of ATP production differs among the genotypes. As expected, in WT neurons, oligomycin caused a complete drop in ATP and IAA had no further effect (Fig. 3C). Remarkably, in Nrf2-KO cells, oligomycin increased the ATP levels (Fig. 3D), which could be attributed to inhibition of ATP hydrolysis by the mitochondrial ATPase (F_1_F_o_-ATPase operating in reverse). Such reversal in F_1_F_o_-ATPase activity towards ATP hydrolysis rather than synthesis could represent an attempt to pump protons across the inner membrane in order to maintain the Δψ_m_, which correlates with the depolarisation seen in TMRM fluorescence after oligomycin (Fig. 1D). In contrast to the apparent lack of inhibition of ATP synthesis by oligomycin, addition of IAA to Nrf2-KO cells produced a fast and complete drop in ATP, further pointing towards reliance on glycolysis for ATP production in Nrf2-deficient cells, in full agreement with the dependence of Δψ_m_ on the presence of glucose in the medium. Such a fast decrease in ATP level in repose to inhibitors of ATP production suggests fast energy consumption in these cells. In Keap1-KD cells, addition of oligomycin resulted in ∼80% drop in ATP, with IAA producing a further ∼20% decrease (Fig. 3E). These results suggest that either Nrf2 deficiency or its constitutive activation reduce the contribution of oxidative phosphorylation towards the synthesis of ATP, and that this effect is especially profound under conditions of Nrf2 deficiency, and are consistent with the increased levels of glycolytic intermediates (G6P, F6P, DHAP, pyruvate, and lactate) that were observed after *NRF2* knockdown (Mitsuishi et al., 2012).

### Nrf2 affects the mitochondrial redox homeostasis

**Fig. 4.**
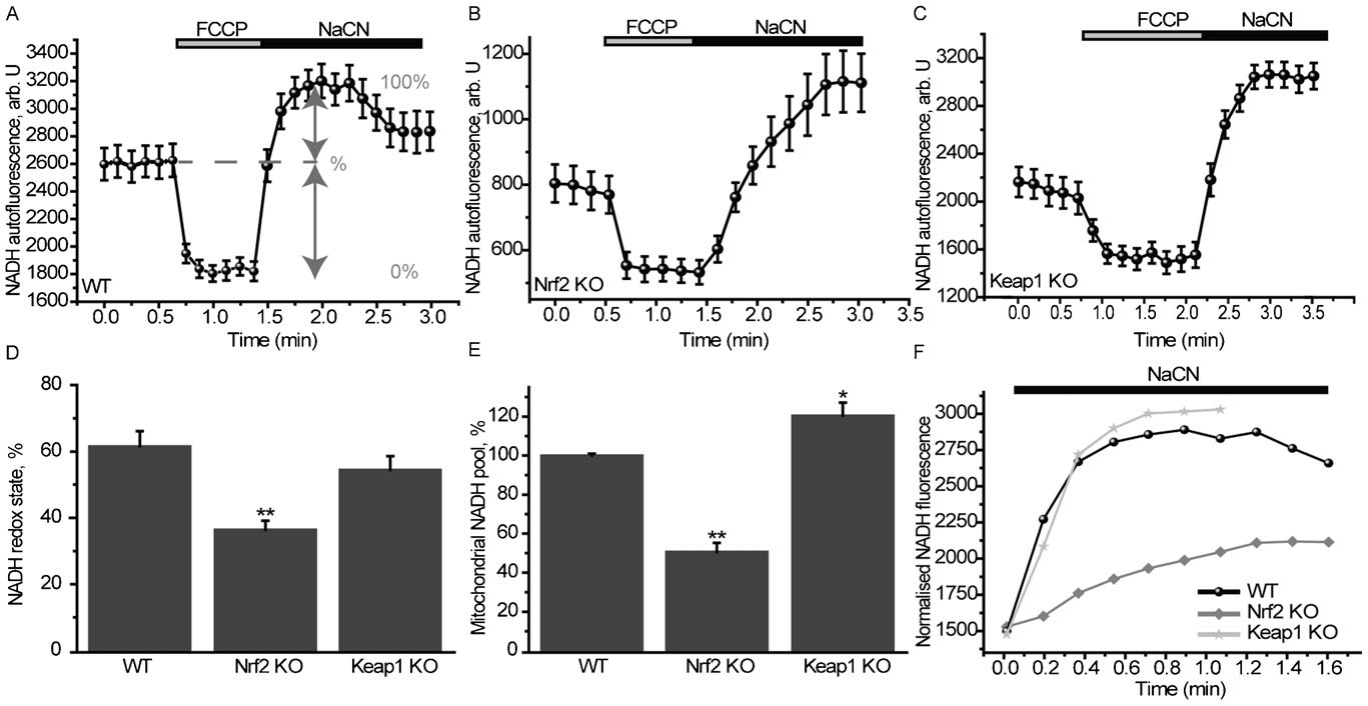
Altered NADH homeostasis in Nrf2-deficient cells. NADH levels were measured using NADH autofluorescence in MEF cells. The redox index was calculated as the basal level relative to maximal respiration after FCCP (1 µM) (0%) and inhibited respiration after NaCN (1 mM) (100%). The NADH pool was expressed as absolute values between maximal and minimal respiration in WT (**A**), Nrf2-KO (**B**) and Keap1-KO (**C**) cells. (**D**) NADH redox state in WT, Nrf2-KO and Keap1-KO. Data presented as mean ± SEM *P<0.001. (**E**) Mitochondrial NADH pool in WT, Nrf2-KO and Keap1-KO. Data presented as mean ± SEM *P<0.05 **P<0.0001. (**F**) The recovery time of the NADH pool after NaCN in WT, Nrf2-KO and Keap1-KO MEFs.

The role of the Keap1-Nrf2 pathway in maintaining the cellular redox homeostasis by controlling the expression of genes encoding enzymes involved in the synthesis, utilization and regeneration of glutathione, thioredoxin and NADPH is well recognized (Greco et al., 2011; Kirby et al., 2005; Lee et al., 2003a; Thimmulappa et al., 2002). To test the possible involvement of Nrf2 in the maintenance of the mitochondrial redox homeostasis, we determined the levels of NADH and FAD by their autofluorescence. Application of the mitochondrial uncoupler FCCP to cells maximises respiration and completely oxidizes the mitochondrial pool of NADH, manifesting as a decrease in fluorescence (**min**) (Fig. 4A). Conversely, the complex IV inhibitor NaCN suppresses respiration preventing NADH oxidation and allowing the NADH pool to regenerate fully (**max**). The redox index is expressed as a percentage of basal NADH autofluorescence compared to **max** and **min** (**Δ**) (Gandhi et al., 2009). Compared to WT (61.5±4.6%, *n* = 78), the NADH redox index was significantly lower in Nrf2-KO MEFs (36.4±2.7%, *n* = 59, *P*<0.001; Fig. 4B,D), whereas it was unaffected in Keap1-KO cells (54.6±4.1%, *n* = 84; Fig. 4C,D). Remarkably, the rate of regeneration of the pool of NADH after NaCN addition was much slower in Nrf2-KO than the corresponding rate in WT or Keap1-KO cells (Fig. 4F). In full agreement, compared to WT (*n* = 78), the total mitochondrial NADH pool was significantly increased in Keap1-KO (121.4±6.7%; *n* = 84, *P*<0.05), and dramatically decreased in Nrf2-KO cells (51.3±4.7%; *n* = 59, *P*<0.0001; Fig. 4E).

**Fig. 5.**
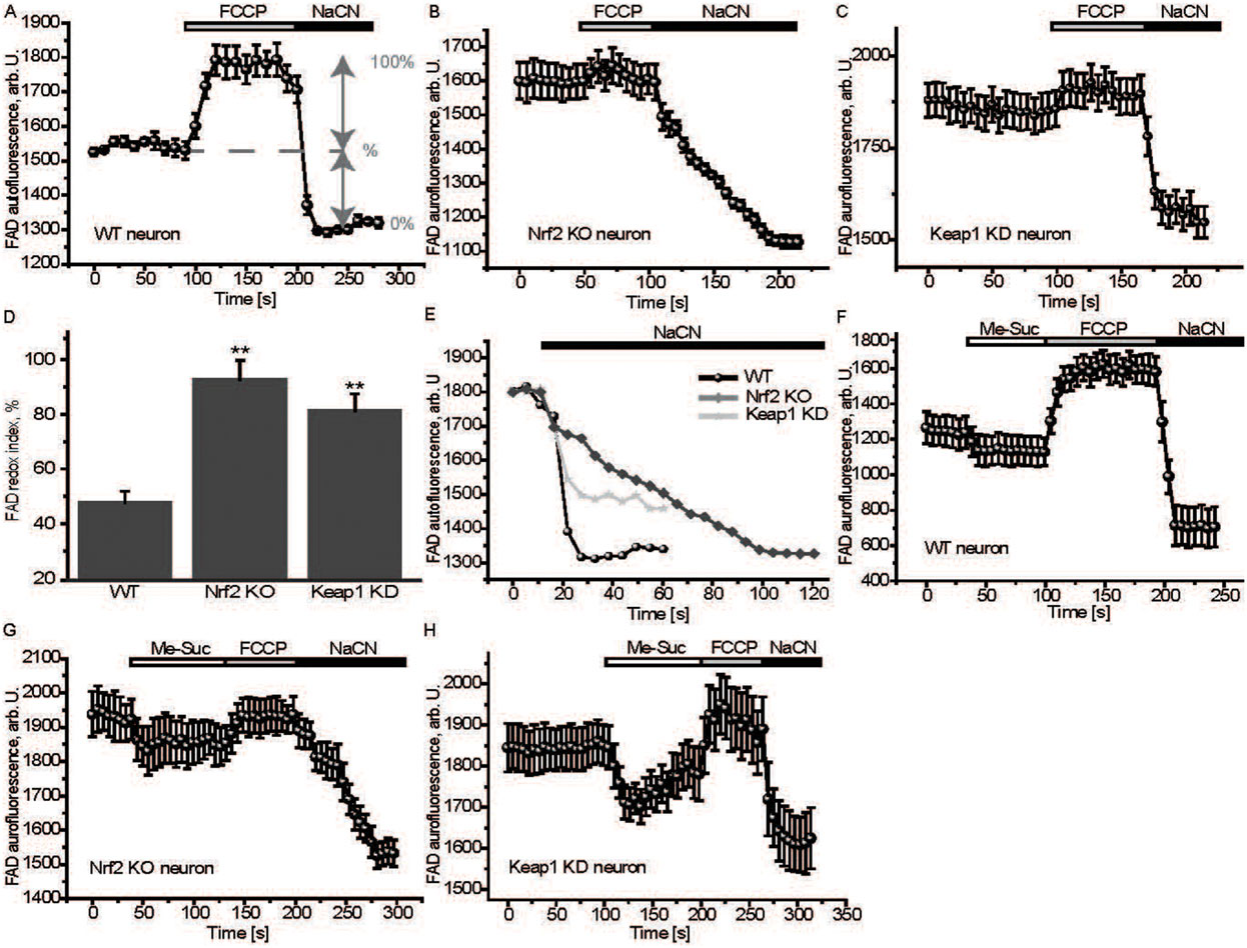
Altered FAD homeostasis in Nrf2-deficient cells. FAD levels were measured using FAD autofluorescence in primary cortical neurons. The redox index was calculated as the basal level relative to maximal respiration after FCCP (100%) and inhibited respiration after NaCN (0%) in WT (**A**), Nrf2-KO (**B**) and Keap1-KD (**C**) neurons. (**D**) FAD redox state in WT, Nrf2-KO and Keap1-KD. Data presented as mean ± SEM *P<0.001. (**E**) The recovery time of the FAD pool after NaCN in WT, Nrf2-KO and Keap1-KD cells. (**F–H**) FAD autofluorescence in WT (F), Nrf2-KO (G) and Keap1-KD (H) cortical neurons after additions of 5 mM methyl succinate, FCCP and NaCN.

The FAD redox index was next assessed; in this case addition of FCCP leads to an increase in fluorescence, whereas NaCN results in a decrease (Fig. 5A). Interestingly, the FAD redox index was higher in both Nrf2-KO (92.6±7.2%, *n* = 91, *P*<0.001; Fig. 5B,D) and Keap1-KD (81.2±6.2%, *n* = 81, *P*<0.001; Fig. 5C,D) neurons compared to WT (47.9±4.1%, *n* = 54; Fig. 5A,D). Similar to the NADH data, the rate of regeneration of FADH_2_ was slower in Nrf2-KO cells compared to their WT and Keap1-KD counterparts (Fig. 5E). Notably, addition of methyl succinate to Nrf2-KO neurons was unable to restore the FAD levels to control values (Fig. 5F,G), whereas in Keap1-KD cells, FAD, upon addition of methyl succinate, fell even below WT values (Fig. 5F,H). This result is consistent with the altered ability of the mutant cells to utilize methyl succinate to maintain Δψ_m_, which is diminished under conditions of Nrf2 deficiency (Fig. 1F), and dramatically accelerated under conditions of constitutive Nrf2 activation (Fig. 1G).

**Fig. 6.**
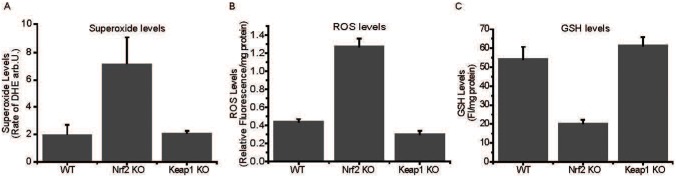
Increased oxidative stress in Nrf2-deficient cells. Levels of superoxide, as measured by dihydroethidium (DHE) (**A**), reactive oxygen species (ROS), as measured by 2′,7′-dichlorodihydrofluorescein diacetate (**B**), and reduced glutathione (GSH) (**C**) in WT, Nrf2-KO and Keap1-KO MEF cells.

**Fig. 7.**
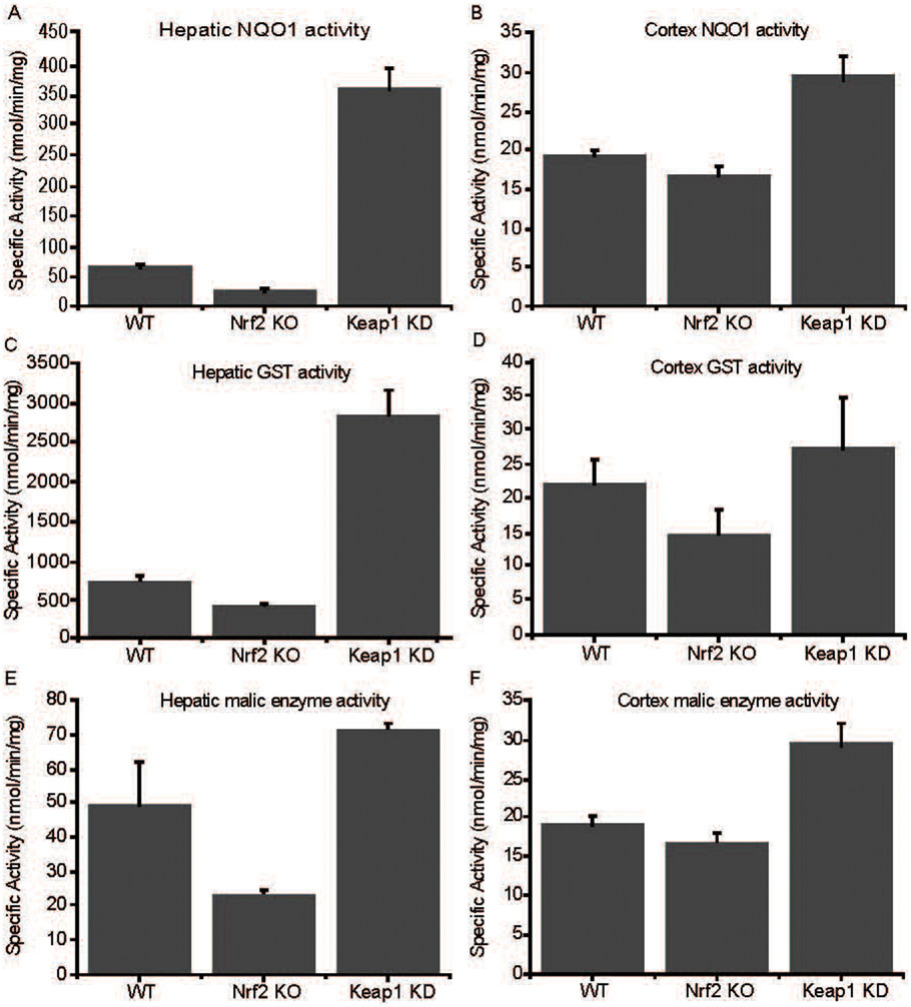
Nrf2-dependent enzyme activities in wild-type, Nrf2- and Keap1-deficient tissues. Hepatic (**A**,**C**,**E**) and cortical (**B**,**D**,**F**) enzyme activities of NQO1 (A,B), GST (C,D), and malic enzyme (E,F). All experiments were performed on tissues isolated from 8-week-old WT, Nrf2-KO, Keap1-KD male mice.

As the mitochondrial function is central to the overall redox homeostasis of the cell, we next determined the levels of superoxide by using the fluorescent probe dihydroethidium in WT, Nrf2-KO and Keap1-KO cells. Consistent with the impaired mitochondrial function in the absence of Nrf2 that was demonstrated by all of the experiments described above, we found that Nrf2-KO have dramatically increased (3–4-fold) levels of superoxide compared to WT cells (Fig. 6A). The overall levels of reactive oxygen species (ROS), as determined by the fluorescence of oxidized 2′,7′-dichlorodihydrofluorescein diacetate, were also higher in Nrf2-deficient cells (Fig. 6B), whereas the levels of reduced glutathione (GSH) were much lower (Fig. 6C). Compared to WT, the activities of three well known Nrf2-dependent enzymes, NAD(P)H: quinone acceptor oxidoreductase 1 (NQO1), glutathione transferases (GSTs), and malic enzyme, in liver and cortex were lower in Nrf2-KO and higher in Keap1-KD mice, confirming the absence and the constitutive activation, respectively of Nrf2 in the transgenic animals (Fig. 7).

## Discussion

The prevailing view of the Keap1-Nrf2 pathway, for which there exists a wealth of experimental evidence, is that it lies at the heart of cellular defense mechanisms and plays a crucial role in adaptation and survival under conditions of stress. It has been implicated as a potential chemoprotection and therapeutical target in a range of diseases, from neurodegenerative disease, chronic kidney disease and diabetes to cancer, and several Nrf2 activators have been or currently are in clinical trials (Calkins et al., 2009; Hayes et al., 2010; Liby and Sporn, 2012; Zheng et al., 2011). Nrf2 is known to act by orchestrating an elaborate genetic program that enhances intrinsic cytoprotective functions, including drug metabolizing, antioxidant, and anti-inflammatory networks. The experiments reported here add another mechanism of cytoprotection to the repertoire of functions of the Keap1-Nrf2 pathway, and demonstrate its profound influence on another line of defense, namely the cell energy metabolism through regulation of the availability of substrates for mitochondrial bioenergetics.

The similar activities of the mitochondrial complexes I, II and IV (Table 1) among the genotypes strongly suggest that the observed defects in respiration and the altered Δψ_m_ in the mutant cells are not due to differences in expression of the genes encoding proteins which form these complexes. Instead, these defects are most likely due to altered availability of substrates for complex I (NADH) and II (FADH_2_). Indeed, in Nrf2-KO cells, the mitochondrial pool of NADH is almost 2-fold lower compared to control cells (Fig. 4E). This could be due to less efficient production of NADH in the TCA cycle as evidenced by the slower rate of regeneration of NADH in response to inhibition of respiration (Fig. 4B,F). This conclusion is further supported by the results from the experiments with substrates for the TCA cycle (glutamate, pyruvate, and malate) which had much smaller effects on the mitochondrial membrane potential and respiration under condition of Nrf2-deficiency. In addition to complex I, Nrf2 deficiency also affects complex II-dependent respiration by limiting the generation of FADH_2_ in the flavoprotein of mitochondrial complex II. Importantly, Nrf2 deficiency leads to activation of glycolysis to compensate for the decreased ATP production due to impairment in oxidative phosphorylation, and also because of the increased ATP demands as a consequence of the use of this energy molecule by the F_1_F_o_-ATPase for the maintenance of Δψ_m_.

The influence on mitochondrial metabolism could partially explain the usefulness of Nrf2 activation in such a wide range of pathological conditions. ATP deficiency has been implicated in several diseases for which Nrf2 activation has been shown to be protective, such as neurodegeneration and diabetes (Burchell et al., 2010; Schuit et al., 2001; Yao et al., 2011), while the switch from oxidative phosphorylation to glycolysis in the presence of oxygen is a hallmark of cancer (Warburg, 1956). The decrease of energy levels in the cells makes them more susceptible to undergo bioenergetics collapse in response to high energy demand activities which eventually may lead to cell death as seen in ischemia and glutamate-induced excitotoxicity (Abramov and Duchen, 2010; Wang et al., 1994). It would also severely impact the functions of the cell as most cellular activities require ATP, from biosynthetic processes to channel activation to kinase activity to signal transduction and cell division (for a review, see Duchen, 2004). Our work highlights the importance of efficient energy metabolism in cytoprotection and implicates Nrf2 as a key regulator of this process.

The most efficient way to produce ATP in the cell is through oxidative phosphorylation. However, increased oxidative phosphorylation comes with the price of increased oxidative stress, as the mitochondria are the major source of ROS in the cell. In the case of Nrf2 activation, this is compensated for by the increase in antioxidant capacity through enhanced expression of genes encoding enzymes such as NQO1, GST, heme oxygenase 1, thioredoxin reductase, and elevated levels of reduced glutathione and thioredoxin (Baird and Dinkova-Kostova, 2011; Hayes et al., 2010). Previous findings have suggested a role for the Keap1-Nrf2 pathway in mitochondrial function; however, until now the underlying mechanisms have been unclear. We now show that one way by which Nrf2 influences mitochondrial function is through the modulation of the utilization of substrates for mitochondrial respiration. Interestingly, the same substrates (NADH and FADH_2_) are important for the function of antioxidant enzymes, whose gene expression is also controlled by the Keap1-Nrf2 pathway (Baird and Dinkova-Kostova, 2011; Hayes et al., 2010). This enforces the reciprocal relationship between oxidative phosphorylation and the redox state of the cell, and the key role of the Keap1-Nrf2 pathway in regulating this balance. The implications of our findings are far-reaching in terms of understanding the multitude of functions of the Keap1-Nrf2 pathway in health and disease.

## Materials and Methods

### Animals

Animal breeding and maintenance were in accordance with the regulations described in the UK Animals (Scientific Procedures) Act 1986. WT, Nrf2 KO, and Keap1 KD C57BL/6 mice were from breeding colonies established at the Medical School Resource Unit, University of Dundee. The animals were kept on a 12-h light/12-h dark cycle, 35% humidity, and were given free access to water and food (pelleted RM1 diet from SDS Ltd., Witham, Essex, UK).

### Cell culture and transfection

Mixed cultures of cortical or midbrain neurons and glial cells were prepared as described (Vaarmann et al., 2010), from mouse pups 1–3 days postpartum. Neurons were easily distinguishable from glia: they appeared phase bright, had smooth rounded somata and distinct processes, and lay just above the focal plane of the glial layer. Cells were used at 14–21 DIV. For measurements of ATP, the cells were transfected for 24 h with the ATP sensing probe AT1.03 (Imamura et al., 2009) using Lipofectamine 2000 according to the manufacturer's instructions.

Mouse embryonic fibroblasts (MEFs) derived from day 13.5 embryos of wild-type, Nrf2-knockout, or Keap1-knockout C57BL/6 mice were maintained in culture dishes coated for 30 min with 0.1% (w/v) gelatin. Cells were grown in Iscoves Modified Dulbecco's Medium (with L-glutamine), supplemented with 10% (v/v) heat-inactivated fetal bovine serum, 1× insulin–transferrin–selenium, and 10 ng/ml epidermal growth factor, all from Gibco–Invitrogen, Paisley, UK. Cultures were maintained at 37°C in a humidified atmosphere of 5% CO_2_ and 95% air.

### Fluorescence measurements

For measurements of Δψ_m_, cells were loaded with 25 nM tetramethylrhodamine methylester (TMRM) for 30 min at room temperature in HBSS (156 mM NaCl, 3 mM KCl, 2 mM MgSO_4_, 1.25 mM KH_2_PO_4_, 2 mM CaCl_2_, 10 mM glucose, and 10 mM HEPES, pH adjusted to 7.35), and the dye was present during the experiment. TMRM is used in the redistribution mode and therefore a reduction in TMRM fluorescence represents Δψ_m_ depolarization. In order to avoid any effects of multi drug resistant pump (MDR) on the level of the indicators in the cells in some experiments we used 10 µM Cyclosporine H or 20 µM verapamil. We have found no difference in results between cells treated with inhibitors of MDR pump and cells without incubation with Cyclosporine H or verapamil. Z-stack images were obtained for accurate analysis. The average intensity of the mitochondrial staining was measured using the Volocity software (PerkinElmer, Waltham, USA). The values for WT were set to 100% and the other genotypes were expressed relative to WT.

Mitochondrial mass was assessed as the percentage of co-localisation of TMRM fluorescence (mitochondria – red) and fluo-4 am (whole cell – green). Cells were loaded with 25 nM TMRM and 5 µM fluo-4 am for 40 min at room temperature. High-resolution z-stack images were obtained. Mitochondrial mass is expressed as the percentage of co-localization of the green (cytoplasmic) signal and the red (mitochondrial) signal. This ratio represents the volume of the cell that is occupied by mitochondria. The co-localization of these signals was set as 100% for WT cells, to enable comparisons among the genotypes.

Confocal images were obtained using a Zeiss 510 uv-vis CLSM equipped with a META detection system and a 40× oil immersion objective. TMRM was excited using the 543 nm laser and fluorescence measured using a 560-nm longpass filter. Fluo-4 was excited using the 488 nm laser and fluorescence measured above 510 nm. NADH autofluorescence was determined with excitation at 351 nm and emission at 375–470 nm. FAD autofluorescence was evaluated with excitation at 458 nm and emission at 520 nm. Illumination intensity was kept to a minimum (at 0.1–0.2% of laser output) to avoid phototoxicity and the pinhole set to give an optical slice of ∼2 µm. ATP levels were measured using AT1.03, a ratiometric construct that undergoes fluorescence resonance energy transfer (FRET) upon contact with ATP. The data were obtained by quantifying FRET after exciting the cyan fluorescent protein at 405 nm (measured at 460 to 510 nm), which in turn excites yellow fluorescent protein, the fluorescence of which is then measured using a bandpass filter from 515 to 580 nm. All data presented were obtained from at least 5 coverslips and 2–3 different cell preparations. Measurements of superoxide using dihydroethidium (DHE) were done as described (Abramov et al., 2010).

### Oxygen consumption

To measure respiration rate in intact cells, approximately 2×10^6^ cells were suspended in HBSS in a Clark-type oxygen electrode thermostatically maintained at 37°C. The oxygen electrode was calibrated with air-saturated water, assuming 406 nmol O atoms/ml at 37°C. Oxygen consumption was measured over time with addition of oligomycin (final concentration 2 µg/ml) and 0.5 µM FCCP.

To measure respiratory control ratio, intact mitochondria were isolated from the brains of WT and Keap1 KD or Nrf2 KO mice by a method of differential centrifugation (Domijan and Abramov, 2011) and resuspended in medium containing 135 mM KCl, 10 mM NaCl, 20 mM HEPES, 0.5 mM KH_2_PO_4_, 1 mM MgCl_2_, 5 mM EGTA at pH 7.1. Oxygen consumption was measured in a Clark-type oxygen electrode thermostatically maintained at 25°C. Glutamate (5 mM) and malate (5 mM) or 5 mM pyruvate were added to measure Complex I-linked respiration, succinate (5 mM) with rotenone (5 µM) were added to measure Complex II-linked respiration. All data were obtained using an Oxygraph Plus system with Chart recording software.

### Mitochondrial complex assays

Activities of respiratory chain complex I, complex II–III (succinate: cytochrome c reductase), complex IV (cytochrome c oxidase: EC 1.9.3.1) and citrate synthase (EC 2.3.3.1; CS) were determined as previously described (Hargreaves et al., 1999). Complex V activity is measured by Blue Native gel electrophoresis (Wittig et al., 2006). In brief, F_1_F_o_-ATPase (Complex V) activity is measured in a reverse direction. ATP is hydrolysed into ADP and Pi (inorganic phosphate). The lead ions in the buffer combine with Pi, which results in the accumulation of lead phosphate, a grey precipitate where the Complex V band is present. Complex V assay is done by incubating the gel overnight in 34 mM Tris, 270 mM glycine, 14 mM MgCl_2_, 6 mM Lead (II) nitrate and 8 mM ATP.

### Determination of ROS

MEFs (200,000 per well) were grown in 0.1% gelatin-coated 6-well plates for 24 h. Cells were washed with PBS and loaded with 1 mL of 10 µM 2′,7′-dichlorodihydrofluorescein diacetate (Invitrogen Ltd, Paisley, UK) in Hank's buffered saline solution (HBSS) for 30 min at 37°C, and generation of reactive oxygen species (ROS) was quantified by the fluorescence intensity of the oxidized probe using a microtiter plate reader with excitation at 485 nm and emission at 530 nm.

### Determination of GSH

Reduced glutathione (GSH) was determined by incubating live cells in Hank's buffered saline solution with 40 µM monochlorobimane (mCB) for 1 h (Rice et al., 1986). The formation of the GS-mCB adduct was quantified using a microtiter plate reader (SpectraMax M2, Molecular Devices) with excitation at 390 nm and emission at 490 nm.

### Enzyme assays

To determine the enzyme activities in mouse organs, portions (∼50 mg) of snap-frozen tissues were pulverized under liquid nitrogen. The resulting powder was resuspended in 10 volumes (0.5 ml) of ice-cold 100 mM potassium phosphate buffer, pH 7.4, containing 100 mM KCl, and complete protease inhibitor cocktail at a dose of one tablet/10 ml buffer. Following mechanical homogenization in an ice bath, the homogenate was subjected to centrifugation at 4°C (15,000 *g* for 10 min). Protein concentrations (Smith et al., 1985) and enzyme activities were determined in the supernatant fractions. The activity of NAD(P)H:quinone oxidoreductase 1 (NQO1) was measured in a coupled colorimetric assay using menadione as a substrate (Prochaska and Santamaria, 1988). Glutathione transferase (GST) activity was measured spectrophotometrically with 1-chloro-2–4 dinitrobenzene (CDNB) as a substrate (Habig and Jakoby, 1981). Malic enzyme activity was determined by monitoring the formation of NADPH using malate as a substrate (MacDonald and Marshall, 2001).

### Statistical analysis

Statistical analysis was performed with Origin 8 (Microcal Software Inc., Northampton, MA, USA) software. Student's t-test was applied. Means expressed ± the standard error of the mean (SEM).
